# Functional conservation of divergent peptidase_M60 O-glycopeptidases in enterococcus

**DOI:** 10.1093/glycob/cwag076

**Published:** 2026-09-07

**Authors:** Liam G Mihalynuk, Benjamin Pluvinage, Olivia Canil, Nicole Thompson, Warren Wakarchuk, Alisdair B Boraston

**Affiliations:** Department of Biochemistry and Microbiology, University of Victoria, PO Box 1700 STN CSC, Victoria, BC V8W 2Y2, Canada; Department of Biochemistry and Microbiology, University of Victoria, PO Box 1700 STN CSC, Victoria, BC V8W 2Y2, Canada; Department of Biochemistry and Microbiology, University of Victoria, PO Box 1700 STN CSC, Victoria, BC V8W 2Y2, Canada; Department of Biological Sciences, University of Alberta, Edmonton, CW 405, Biological Sciences Bldg, Alberta, T6G 2E9, Canada; Department of Biological Sciences, University of Alberta, Edmonton, CW 405, Biological Sciences Bldg, Alberta, T6G 2E9, Canada; Department of Biochemistry and Microbiology, University of Victoria, PO Box 1700 STN CSC, Victoria, BC V8W 2Y2, Canada

**Keywords:** glycoprotein recognition, metallopeptidase, mucinase, *O*-glycopeptidase, structural glycobiology

## Abstract

*O*-glycopeptidases are proteolytic enzymes that obligately recognize the *O*-glycans appended to their substrates. Peptidase_M60 proteins comprise a superfamily of putative metal-dependent *O*-glycopeptidases that were initially described in host-associated bacteria but are now known to be distributed across bacteria occupying both host-associated and environmental niches. Although several members of this superfamily have been shown to possess *O*-glycopeptidase activity, the family is highly divergent at the amino acid sequence level, making it unclear whether this activity is conserved across all members. Here, we show that two peptidase_M60 enzymes, EfmM60 and EfcM60, from strains of *Enterococcus faecium* and *Enterococcus faecalis*, respectively, which are only distantly related at the primary sequence level to previously characterized *O*-glycopeptidases, exhibit both mucinase and *O*-glycopeptidase activity. Structural analysis of EfmM60 reveals distinct active-site features relative to previously characterized peptidase_M60 enzymes that provide a molecular basis for its ability to accommodate extended and branched *O*-glycans. Together, these findings highlight functional conservation within a highly divergent peptidase_M60 family and suggest that enterococcal *O*-glycopeptidases may contribute to ecological versatility by enabling access to *O*-glycosylated substrates across diverse biological contexts.

## Introduction

Mucus is one of the first barriers encountered by colonizing or invading bacteria (Linden et al. 2008; Fyderek et al. 2009). The main structural components of mucus are mucins, high molecular weight glycoproteins with an extended protein backbone that is heavily decorated with heterogeneous glycan units (Linden et al. 2008). *O-*glycosylation of mucins occurs on serine or threonine residues in characteristic proline, threonine and/or serine (PTS) rich repeats found in all mucins and mucin domains (Johansson et al. 2011). Elaboration of the priming α-D-GalNAc through different glycosyltransferases results in the eight core glycan structures, the first four of which are common in humans (Brockhausen and Stanley 2015). These structures form the base on which more complex mucin glycans can be built.

Microorganisms ranging from commensals to pathogens employ diverse strategies to overcome mucus barriers. Carbohydrate-active enzymes (CAZymes) are a well-understood mechanism to degrade the glycan portions of these molecules; however, enzymes that directly cleave the protein backbone of mucins have also recently been discovered (Nakjang et al. 2012; Noach et al. 2017). Members of the peptidase_M60 family specifically recognize *O-*glycosylation and subsequently cleave the peptide backbone immediately N-terminal to the glycosylated residue; non-glycosylated proteins are not hydrolyzed (Noach et al. 2017; Pluvinage et al. 2021). The nomenclature of *O*-glycopeptidase active sites utilizes the classic subsite nomenclature of Schecter and Berger while incorporating the addition of “G-sites” to describe the recognition of the glycan components on their substrates (Hooper 2002; Noach et al. 2017; Pluvinage et al. 2021). Genes encoding peptidase_M60 proteins are frequently associated with host-adapted microbes and in some cases a link between their expression and erosion of the host mucosal layers has been made (Desai et al. 2016).

The peptidase_M60 enzymes characterized in several recent studies appear to fall into three broad classes with respect to the accommodation of glycans in the active site (Noach et al. 2017; Shon et al. 2020; Pluvinage et al. 2021, 2026; Chongsaritsinsuk et al. 2023; Narimatsu et al. 2026). The first class is of enzymes that specialize in linear glycans, either Tn (α-linked O-GalNAc), Core 1 (C1, Galβ1-3GalNAcα-), and extended C1 structures. This may include activity on Core 3 (C3, GlcNAcβ1-3GalNAcα-) but activity on this glycan is less well established. Notable examples are BT4244 from *Bacteroides thetaiotaomicron* and all of the characterized peptidase_M60 enzymes from *Akkermansia muciniphila* (AMUC_0627, AMUC_0908, and AMUC_1514)(Noach et al. 2017; Shon et al. 2020, 2022; Taleb et al. 2022; Wardman et al. 2023; Ndeh et al. 2025). The second class is of enzymes that are active on linear glycans and glycans that are branched at O6 of the core α-linked O-GalNAc, such as Core 2 (C2, Galβ1-3[GlcNAcβ1–6]GalNAcα-), 2,6-sialyl Core 1 (6SC1, Galβ1-3[Neu5Acα2-6]GalNAcα-), and more elaborate sialylated versions of these. This may include activity on 6-sialyl Tn (6STn, Neu5Acα2-6GalNAcα-) but activity on this glycan is also less well established. Well characterized examples include BcM60C and BcM60K from *Bacteroides caccae* and SmE from *Serratia marcescens* (Chongsaritsinsuk et al. 2023; Pluvinage et al. 2026). The third general class is enzymes that appear to require a glycan that is modified at O6 of the core α-linked O-GalNAc (i.e. they lack activity on linear glycans). To date these are the rarest enzymes, currently being limited to ZmpA and ZmpC from *Clostridium perfringens* (Noach et al. 2017; Pluvinage et al. 2021). All classes of the enzymes possess a G1’ subsite that accommodates the core Tn motif and a G2’ subsite that enables binding of linear glycans. Class 2 and class 3 enzymes are characterized by the presence of an open G2” subsite in which a sugar O6-linked to the core GalNAc can sit (Noach et al. 2017; Pluvinage et al. 2026).

Despite growing structural and biochemical insight into peptidase_M60 enzymes, it remains unclear how extensively O-glycopeptidase activity is conserved among highly divergent family members and how variation in their active sites influences O-glycan recognition. Enterococci occupy a broad range of host-associated and environmental niches and encode putative peptidase_M60 proteins that are divergent from those that are currently characterized, providing an opportunity to examine the relationship between sequence divergence, substrate recognition, and catalytic function. *Enterococcus faecium* and *Enterococcus faecalis* are common members of the mammalian microbiota and important opportunistic pathogens (Billström et al. 2008; Fisher and Phillips 2009; Lebreton et al. 2014). Given their frequent association with mucosal and other glycoprotein-rich environments, we investigated two highly divergent peptidase_M60 proteins encoded by strains of these species. We show that their recombinant metallopeptidase domains, EfmM60 (from *E. faecium*) and EfcM60 (from *E. faecalis*), possess metal-dependent mucinase and O-glycopeptidase activity. Structural analysis of EfmM60 provides a molecular basis for its ability to accommodate extended and branched O-glycans.

## Results

### Distribution of peptidase_M60 homologs across enterococcal species and niches

We initially identified putative enterococcal peptidase_M60 proteins by querying the InterPro database for members of the peptidase_M60 family (IPR0361161), which at the time included entries from *E. faecium* and *E. faecalis*. Using BLASTP searches with the metallopeptidase domains of EfmM60 and EfcM60 as queries we identified additional divergent homologs not captured by InterPro annotations. These were filtered to enterococcal species and amino acid sequence identity to >30%, resulting in a data set of over 1000 hits spanning 30% to 100% amino acid sequence identity with the query sequences. Given the large number of hits and significant number of different enterococci, we thought to assess the possible ecological distribution of enterococcal peptidase_M60 homologs in this genus. To do this, we used our list of hits to survey the origin of the enterococcal species in which at least one copy of a putative peptidase_M60 homolog was found in at least one strain. The results included enterococci that were clinically isolated or human-associated, as well as isolates from birds, reptiles, insects, livestock, aquatic environments, and plant-associated habitats, indicating broad ecological distribution (Supplementary Table 1).

Because of their frequent association with humans, we focused on putative peptidase_M60 O-glycopeptidases from *E. faecium* and *E. faecalis.* Specifically, we concentrated on locus tag MDV4556911 from *E. faecium* strain 51271882, which was isolated from human blood, and locus tag HCR3094972 from *E. faecalis* strain BE16, which was isolated from human saliva. MDV4556911 and HCR3094972 are 1521 and 1010 amino acid proteins, respectively. AlphaFold 3 generated models of these proteins revealed quite extensive multimodularity (Supplementary Fig. 1A and B). The predicted metallopeptidase domains show only ~30% amino acid sequence identity to one another. In the classification of Pluvinage et al. (2026) the metallopeptidase domain of MDV4556911 has 100% sequence identity to A0A7V7KSJ4, also from *E. faecium*, placing this protein in clade 13 of the classification. HCR3094972 was also in clade 13 with the closest homolog of 43% amino acid sequence being A0A0R2DS38 from *Holzapfeliella floricola* DSM 23037. Also in clade 13, with ~30% identity to both of the enterococcal proteins, is SmE from *S. marcescens.* This peptidase_M60 O-glycopeptidases has been characterized by glycoproteomics and shown to have broad specificity for linear and O6-branched glycans (Chongsaritsinsuk et al. 2023). No member of clade 13 has yet been structurally characterized and members this clade generally show less than ~20% sequence identity with characterized peptidase_M60 enzymes from other clades. Nevertheless, both enterococcal proteins had the overall predicted fold characteristic of peptidase_M60 O-glycopeptidases and conservation of the WXXXHEXXH motif that contributes the zinc binding catalytic machinery and O-linked GalNAc binding in the active site.

Though the strains we focused on originate from human hosts, an examination of the origins of the strains containing orthologs of MDV4556911 revealed broad distribution of the *E. faecium* strains containing them, including shellfish, fish, humans, pigs, cows, wild birds, and wild boar. Likewise, orthologs of WP_104875352 were also found in *E. faecalis* strains with wide distribution, including humans (umbilicus, feces, saliva), cow (including retail ground beef), market fowl (chicken, duck, turkey), marine environments, and wastewater. Thus, even within individual enterococcal species, peptidase_M60–encoding strains are distributed across markedly diverse host-associated and environmental niches, indicating that these enzymes are not restricted to human colonization but may be broadly associated with enterococcal ecological versatility.

To interrogate the activity of the putative *E. faecium* and *E. faecalis* O-glycopeptidases identified above we produced and purified the metallopeptidase domains in isolation for biochemical analysis. These domains are hereafter referred to as EfmM60 and EfcM60 for the *E. faecium* and *E. faecalis* proteins, respectively.

### Metal-dependent mucinase activity of enterococcal peptidase_M60 proteins

**Figure 1:**
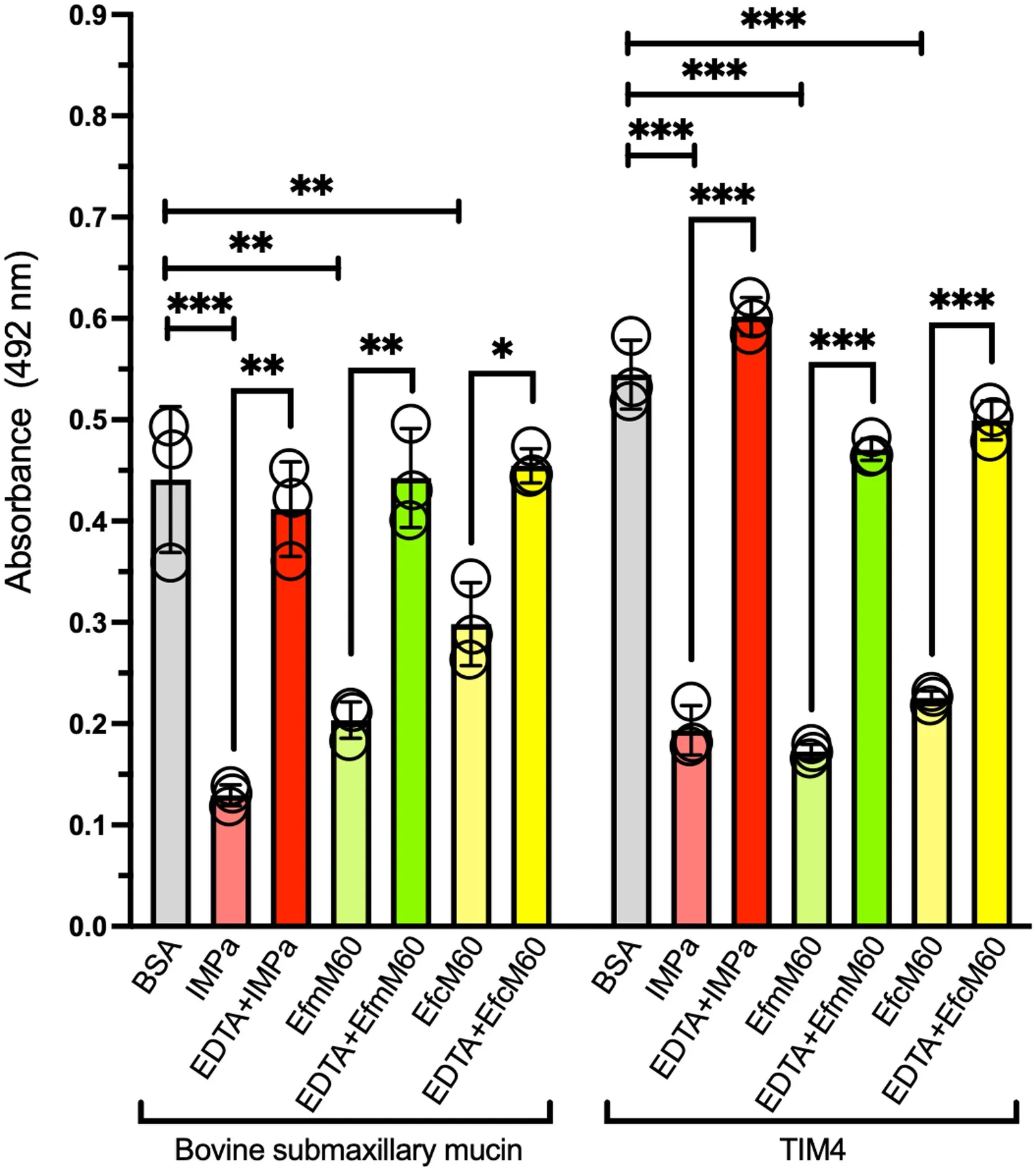
Mucinase assay showing the activity of EfmM60 and EfcM60 on bovine maxillary mucin (BSM). A reduction in signal indicates loss of immobilized biotinylated BSM and therefore mucinase activity. IMPa was included as a positive control and BSA as a negative control. P-value symbols are as follows: *P* < 0.05 (^*^), *P* < 0.01 (^**^), *P* < 0.001 (^***^), *P* < 0.0001 (^****^). EDTA treated samples were not significantly different (i.e. *P* > 0.05) than the corresponding no enzyme, BSA control.

The mucinase activities of EfmM60 and EfcM60 were assessed using an established mucinase assay with immobilized, biotinylated bovine submaxillary mucin (BSM) and T-cell membrane 4 (TIM4) as representative of mucin and non-mucin O-glycosylated substrates. Mucinase activity results in removal of the biotinylated substrates from the micro-titre plate and a corresponding reduction in the signal upon biotin detection, as observed for the IMPa positive control (Fig. 1). Both EfmM60 and EfcM60 were active on BSM and TIM4; however, treatment with EDTA reduced activity to that of the no-enzyme condition, consistent with metal-dependent peptidase activity (BSA negative control) (Fig. 1). These results demonstrate that both proteins possess metal-dependent mucinase activity.

### EfmM60 and EfcM60 exhibit O-glycan–dependent proteolytic activity

To establish the glycan dependence of EfmM60 and EfcM60 O-glycopeptidase activity and assess general selectivity we used a FRET-based activity screen (Canil et al. 2026; Pluvinage et al. 2026). The eleven amino acid linker separating the fluorescent protein domains of our FRET substrates represented twenty-four different PTS repeats from fourteen common mucins and three non-mucin proteins.

**Figure 2:**
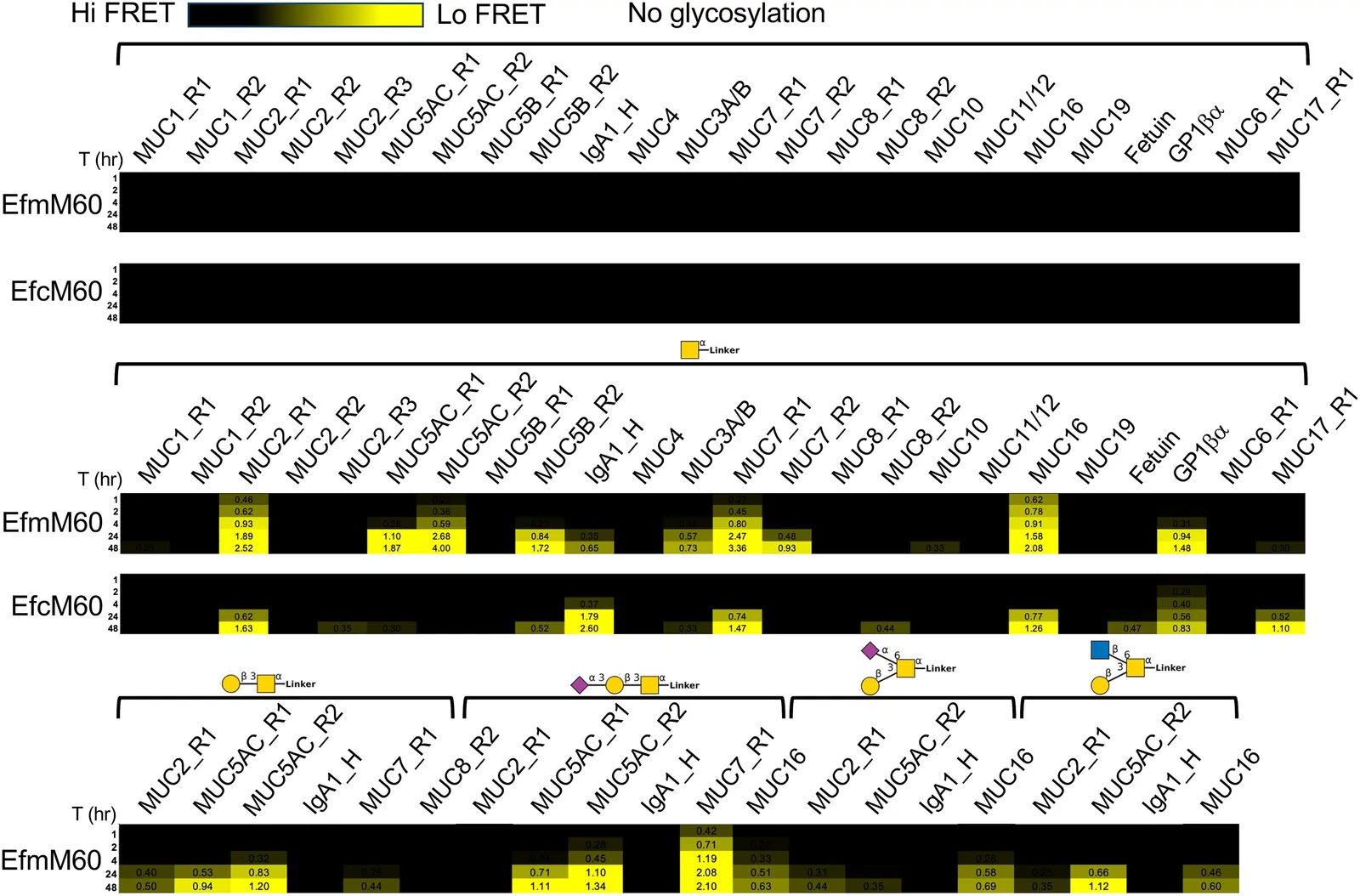
*O*-glycopeptidase activity revealed by a custom screen of mucin-like FRET substrates. Heat maps show FRET values (calculated by the ratio-of-ratios approach) over time (indicated to the left of the heat maps). The coloring is ramped from a low threshold of 0.2 to a high of 1.0. Increasing FRET indicates substrate cleavage.

Both proteins were screened on the first-generation substrate array containing unglycosylated substrates and substrates bearing only Tn antigen. Neither protein showed activity on unglycosylated substrates, whereas both cleaved Tn modified substrates, as revealed by the decrease in FRET signal (Fig. 2), confirming *O*-glycan dependent proteolytic activity. Cleavage positions were not directly mapped in this study but are inferred to occur N-terminal to the glycosylated residue based on established peptidase_M60 activity (Noach et al. 2017; Shon et al. 2020). EfmM60 showed detectable activity on several substrates, including MUC2_R1 and MUC16, within the first hour of reaction. EfcM60 exhibited consistently lower apparent cleavage rates across the substrate panel but nevertheless retained clear glycan-dependent activity on a similar subset of substrates, consistent with functional conservation despite substantial sequence divergence. The only notable difference between EfmM60 and EfcM60 was the negligible activity of EfcM60 on the MUC5AC linkers. Overall, compared to other peptidase_M60 enzymes characterized by this method, both EfmM60 and EfcM60 have restricted activity on the repertoire of Tn modifed substrates cleaving only 10 and 8 of the substrates, respectively. BT4244, BcM60K, BcM60C, and BcM60M cleaved 16–18 of these substrates (Pluvinage et al. 2026). This likely represents greater specificity of EfmM60 and EfcM60 for specific features of the peptide backbone. The substrate repertoires of EfmM60 and EfcM60 were largely represented within those of the prior characterized Bacteroides proteins. However, the enterococcal enzymes displayed activity on the IgA1_H linker, which only BT4244 was active on in this assay. Cleavage of the IgA1 hinge is potential mechanism to inhibit host immune response at a mucosal surface. Though activity on the O-glycosylated linker sequence of IgA1 is only documented for very few peptidase_M60 enzymes and the significance of this is not yet clear (Noach et al. 2017; Ndeh et al. 2025; Pluvinage et al. 2026).

Given the lower apparent activity of EfcM60, subsequent mechanistic and structural analyses focused on EfmM60 as a representative enzyme with more robust catalytic properties. We further screened EfmM60 with a second generation substrate array containing substrates modified with C1, 3SC1 (2,3-sialyl Core 1, Neu5Acα2-3Galβ1-3GalNAcα-), C2, and 6SC1 glycans. EfmM60 cleaved the same linker sequences when they were modified with C1, 3SC1, 6SC1, or C2 glycans (Fig. 2), indicating the ability to accommodate larger linear and glycans that are branched on the O6 of the core GalNAc. However, while the rate of signal generation for C1 and 3SC1 substrates was similar to that of the Tn modified versions, the rate of signal production was generally slower for the 6SC1 and C2 modified substrates, suggesting lower activity. This places EfmM60 into the second general class of peptidase_M60 O-glycopeptidases that accommodate branched glycans.

To quantify the catalytic efficiency of EfmM60 on representative model substrates and provide context for comparison with previously characterized *O*-glycopeptidases, we performed steady-state kinetic analyses with Tn modified MUC2_R1 and MUC16 linkers. To assess internal consistency, kinetic parameters were obtained both from global fits of the complete dataset and from separate global fits of each enzyme concentration, which did agree within relatively narrow 95% confidence intervals (Table 1, Supplementary Fig. 2). EfmM60 had approximately twice the catalytic efficiency (k_cat_/K_M_) on MUC16 compared with MUC2_R1. Given the similar K_M_ values, the difference in k_cat_/K_M_ comes largely from the 2-fold slower rate of turnover (k_cat_) on MUC2_R1. These k_cat_ values were ~ 5–10-fold slower than the determination for different *O*-glycopeptidases on *O*-glycopeptide-based FRET substrates with chemical fluorophores (Pluvinage et al. 2021). By virtue of small K_M_ values, the k_cat_/K_M_ values for EfmM60 were ~ 2–30-fold larger than observed in prior studies with synthetic substrates; however, they were similar to those observed for the peptidase_M60 *O*-glycopeptidase BT4244 and O-glycopeptidases from *B. caccae* on similar fluorescent protein-based *O*-glycosylated substrates (Pluvinage et al. 2026). These values represent apparent kinetic parameters for the isolated EfmM60 metallopeptidase domain acting on defined model substrates and should not be extrapolated directly to the full-length enzyme acting on native glycoproteins in situ. The physiological significance of the slow apparent turnover will depend on factors including enzyme abundance, localization, substrate presentation and the accessibility of productive cleavage sites.

**Table 1:** Kinetic parameters of EfmM60.

**Data set**	**Parameter (units)**	**MUC16 linker with Tn**				**MUC2 linker with Tn**			
		**Value**	**Standard** **Error**	**CV%**	**+/− 95%**	**Value**	**Standard** **Error**	**CV%**	**+/− 95%**
	k_cat_/K_M_ (min^−1^ mM^−1^)	211	5.9	6.5	23	106	1.5	5.0	8
All data	k_cat_ (min^−1^)	0.20	0.004	4.0	0.01	0.12	0.003	2.9	0.01
	K_M_ (μM)	0.95	0.03	NA	0.13	1.11	0.02	NA	0.09
	k_cat_/K_M_ (min^−1^ mM^−1^)	248	14	13	41	173	9.1	18	39
0.1 mM EfmM60	k_cat_ (min^−1^)	0.20	0.01	8.1	0.02	0.12	0.004	9.1	0.01
	K_M_ (μM)	0.80	0.07	NA	0.20	0.69	0.06	NA	0.26
	k_cat_/K_M_ (min^−1^ mM^−1^)	209	8.4	9.0	28	104	1.8	5.9	9
0.2 mM EfmM60	k_cat_ (min^−1^)	0.21	0.004	5.3	0.02	0.12	0.004	3.3	0.01
	K_M_ (μM)	1.00	0.05	NA	0.16	1.14	0.02	NA	0.10

### Structural basis for accommodation of extended O-glycans by EfmM60

**Figure 3:**
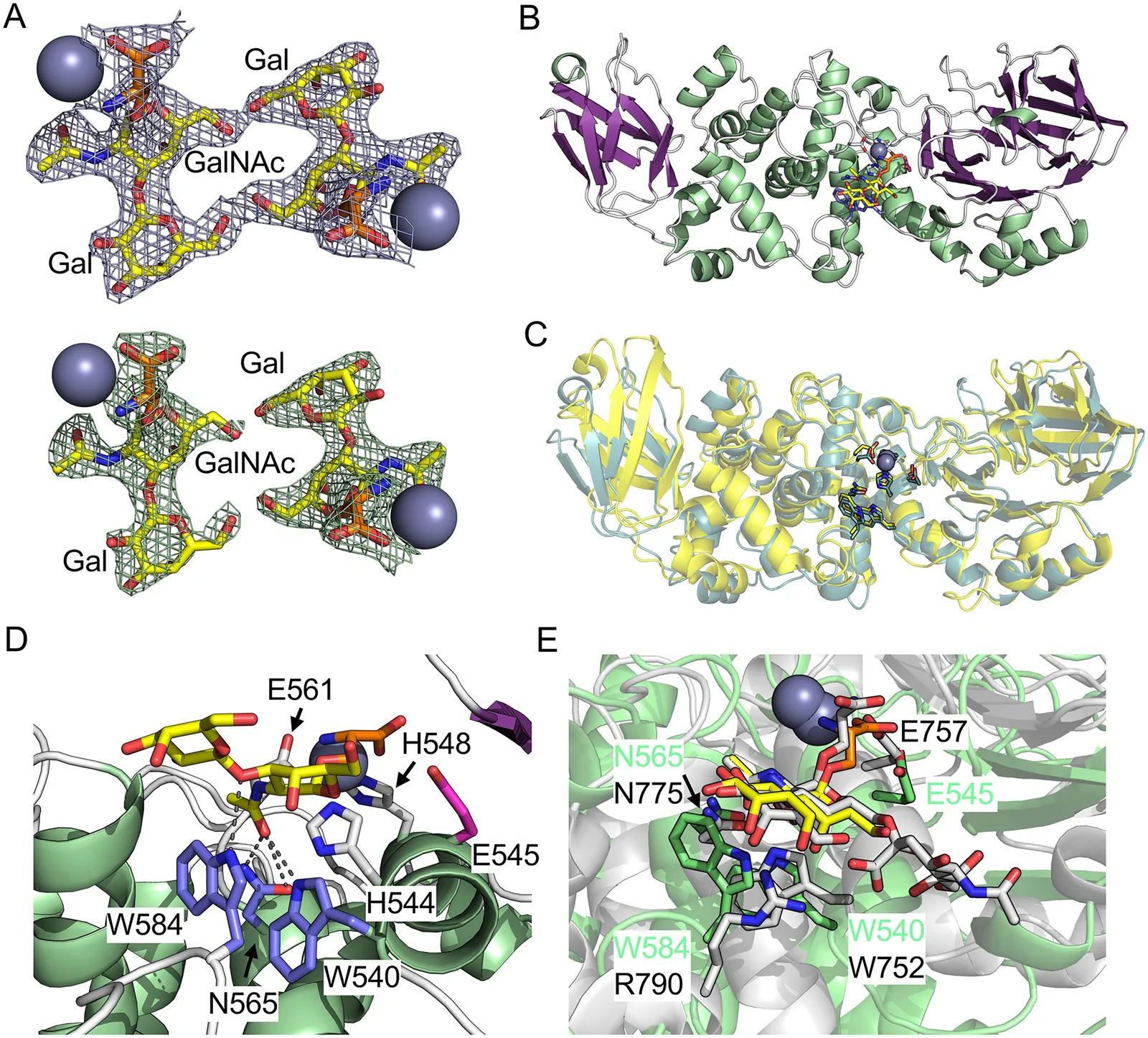
Structural analysis of EfmM60. A) Electron density shown as mesh of each C1-Ser ligands bound to the two EfmM60 monomers in the asymmetric unit. The top image shows the 2F_o_-F_c_ map contoured at 1σ in blue mesh and the bottom shows the F_o_-F_c_ map contoured at 3σ in green mesh. B) Cartoon representation of an EfmM60 monomer with the bound C1-ser ligand. Ligand binding residues are shown as sticks. The thermolysin-like domain is shown in green while the structural Ig-like domains are shown in purple. C) Overlap of the EfmM60 crystal structure (green) with the AlphaFold 3 model of EfcM60 (yellow). Conserved substrate and zinc binding residues are shown as sticks. D) Closeup of the EfmM60 active site. Potential hydrogen bonds are shown as dashes. E) Overlap of EfmM60 (green with C1-ser colored yellow and orange) with ZmpB in complex with 6SC1 (grey, PDB ID 5KDU). Substrate binding residues and the catalytic glutamates are shown as sticks.

The ability of EfmM60 to cleave substrates bearing extended and branched *O*-glycans suggested an active site architecture distinct from previously characterized peptidase_M60 enzymes, motivating structural analysis. We crystallized EfmM60 and determined its structure by X-ray diffraction to 2.3 Å resolution in complex with serinyl-Core 1 (C1-ser, Galβ1–3GalNAcα1-Ser or T-antigen). The electron density of the C1-ser was of sufficient quality to model the two ligands in the asymmetric unit, one bound to each monomer of EfmM60 (Fig. 3A). All of the residues from residues 307–816 could be modeled in both monomers of EfmM60 in the AU except for residue S402 in monomer B.

The overall fold of EfmM60 is that common to peptidase_M60 enzymes with an α-helical thermolysin-like catalytic core flanked by two structural Ig-like domains (Fig. 3B)(Noach et al. 2017; Taleb et al. 2022). The comparison of AlphaFold 3 model of EfcM60 to the crystal structure of EfmM60 showed the predicted structural components and 3-dimensional position of them in EfcM60 was very similar to that of EfmM60 with a root mean square deviation (RMSD) of 1.7 Å (Fig. 3C). Moreover, key features of the predicted active site were well conserved. We used FoldSeek to search more widely for structures similar to EfmM60, which identified the catalytic domain of *C. perfringens* ZmpB (PDB ID 5KDS) as the most structurally similar protein in the Protein Data Bank. However, even truncating the EfmM60 coordinates to just the most conserved α-helical core resulted in a relatively poor RMSD of 5.8 Å with ZmpB. EfmM60, and by extension EfcM60, thus display mucinase and *O*-glycopeptidase activity as well as the general structural features of peptidase_M60 *O*-glycopeptidases but are quite distantly related at the primary and overall structural levels to known peptidase_M60 enzymes.

Potential hydrogen bonding in the active site of EfmM60 was mainly confined to the GalNAc residue in the G’ site (Fig. 3D). We refined our structural overlap with the 6SC1 complex of ZmpB (PDB ID 5KDU) by using the bound GalNAc residues as reference points for the superposition. This revealed structurally conserved hydrogen bonding between the GalNAc residue and an active site asparagine, the tryptophan on the catalytic helix, and an absence of interactions between the protein and the β-1,3-linked galactose (Fig. 3E). R790 in ZmpB is conserved amongst currently characterized peptidase_M60 *O*-glycopeptidases, where it hydrogen bonds with the axial O4 of the GalNAc residue. In EfmM60 (and EfcM60) this residue is replaced by a tryptophan residue where the indole nitrogen hydrogen bonds with oxygen of the glycosidic bond joining the GalNAc residue to the galactose. Presumably this hydrogen bond is maintained with the O3 hydroxyl when binding the Tn antigen. The conserved constellation of catalytic residues, zinc binding residues, and key glycan binding residues, along with the position of the ligand in the EfmM60 active site is completely consistent with hydrolysis of the peptide bond immediately N-terminal to the site of glycosylation, a property observed for all other members of the peptidase_M60 family to date.

**Figure 4:**
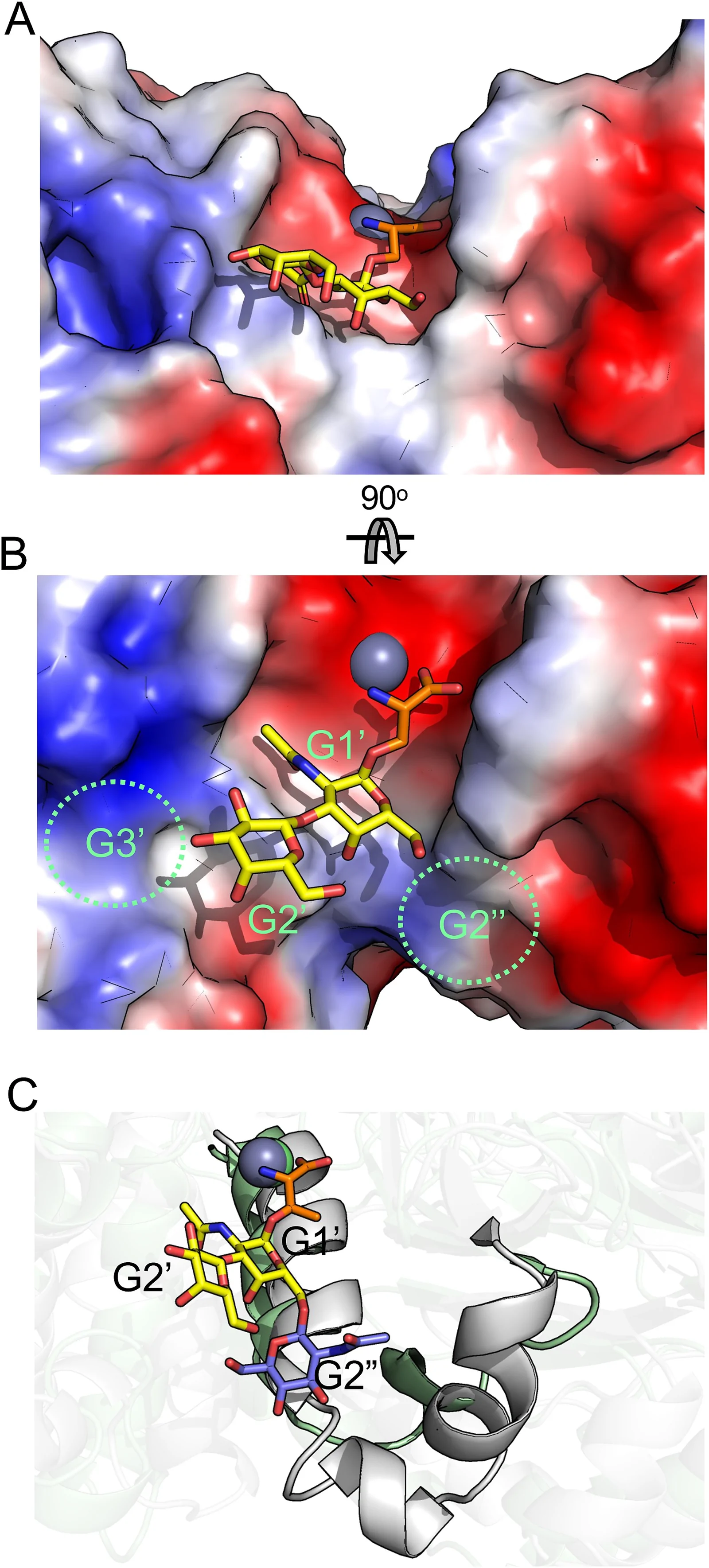
Glycan binding subsites of EfmM60. A and B) Surface representations of the active site of EfmM60. Glycan binding subsites and putative glycan binding subsites are indicated. Zinc atoms are shown as grey spheres in all panels. C) Overlap of BcM60C in complex with C2-Threonine (PDB ID 9PLA)(grey with the ligand shown as sticks) and EfmM60 (green). The cataltyic zinc atoms are shown as spheres adjacent to the helix running down the Centre of the catalytic site.

The active site of EfmM60 is quite shallow and open (Fig. 4A). The region where a residue on O6 of the GalNAc residue opens out into solvent with space to accommodate branched glycans via a G2” site (Fig. 4B). Similarly, there is space for a G3’ site to accommodate extensions on the O3 of the galactose, such as α-2,3-Neu5Ac. To probe this in more detail we overlapped the EfmM60 structure with the C2 complexed structure of BcM60C from *B. caccae* (Supplementary Fig. 3A) (Pluvinage et al. 2026). This further supported the potential presence of a G2” subsite, consistent with the cleavage of substrates bearing C2 and 6SC1 glycans. Extrapolation from O2 of the terminal galactose residue also suggests a G3’ subsite that comprises a surface patch of basic residues that may aid in recognizing the terminal sialic acid of 3SC1 (Supplementary Fig. 3A). Together, these structural features provide a molecular explanation consistent with the ability of divergent enterococcal peptidase_M60 enzymes to accommodate a wide range of *O*-glycan elaborations, consistent with their occurrence across enterococci inhabiting diverse host-associated and environmental niches.

## Discussion

Enterococcus is a genus comprising approximately 38–40 recognized species that occupy a remarkable range of ecological niches, including the gastrointestinal tracts of mammals, reptiles, fish, and insects, as well as plants, soil, surface waters, and fermented foods (Campos et al. 2006; Vu and Carvalho 2011; Lebreton et al. 2014). This ecological breadth suggests a long evolutionary history that includes both host association and environmental persistence, potentially extending back hundreds of millions of years to early vertebrate–invertebrate divergence and positioning enterococci among the earliest bacteria to repeatedly interface with animal-associated habitats (Gilmore et al. 2013).

In this context, the broad but non-universal distribution of peptidase_M60 homologs across enterococci is notable. Rather than being restricted to clinical isolates or human-associated strains, peptidase_M60–encoding enterococci are found across species and strains inhabiting diverse host-associated and environmental niches. This species- and strain-level ecological breadth suggests that *O*-glycopeptidases are not specialized virulence factors, but rather evolutionarily flexible enzymes that may enable access to *O*-glycosylated substrates across both host-associated mucosal surfaces and, potentially, saprophytic environments enriched in complex glycoproteins. In such environments, the ability to cleave *O*-glycosylated proteins may facilitate nutrient acquisition independent of direct host association. Accordingly, mucins are plausible substrates in host-associated mucosal environments, whereas other O-glycoproteins may be relevant in different host-associated or environmental settings; the physiological substrates of these enzymes are therefore likely to be context dependent.

Despite sharing very low amino acid sequence identity with previously characterized peptidase_M60 enzymes, both EfmM60 and EfcM60 were shown to be bona fide *O*-glycopeptidases, though EfcM60 displayed consistently lower apparent catalytic rates under the conditions tested. This may reflect intrinsic differences in catalytic efficiency, preference for features not represented in the substrates used, or regulatory features that are not captured in the recombinant metallopeptidase domain alone, including potential contributions from non-catalytic domains in the full-length protein. Regardless, the detection of glycan-dependent activity in both enzymes supports the conclusion that *O*-glycopeptidase function is conserved despite substantial sequence divergence, consistent with strong selective pressure to maintain *O*-glycan–dependent proteolysis while permitting substantial structural variation.

The ability of EfmM60 to cleave substrates bearing extended and branched *O*-glycans further suggests that functional success across diverse ecological settings may depend less on strict glycan specificity than on the capacity to accommodate a range of *O*-glycan elaborations. Structural analysis of EfmM60 revealed an open and permissive active site architecture that provides a molecular basis for this glycan tolerance (Fig. 3F and G). While we have suggested the presence of a G2” subsite that enables accommodation of glycans branded at O6 of the core α-O-GalNAc a more accurate description, perhaps, is that this region of the active site is so open that the subsite is potentially entirely absent. The presence of a G2” subsite is in most cases, but not all, correlated with shortening of the central active site α-helix that contains the catalytic residues. In EfmM60, not only is the active site a-helix suitably shortened to open a G2” subsite but the loop exiting from this helix is also shortened, making this region of the active site even more open (Fig. 4C). The implications are that this region of the glycan binding site likely contributes minimal interactions with the substrate. Further, the highly open nature in this region may also provide the potential to accommodate different kinds of glycan extensions on the O3 or O4 of the GlcNAc residue on C2. The surface features in the AlphaFold 3 models of EfcM60 and SmE also have notably open glycan binding sites very similar to that of EfmM60, consistent with the observed ability of SmE to cleave mucin-like substrates with a variety of O6 modified glycans (Supplementary Fig. 3B and C) (Chongsaritsinsuk et al. 2023). Overall, this offers an explanation for how divergent peptidase_M60 enzymes may remain functional across environments in which glycoprotein structures vary widely through recontouring of active sites. Further, these findings suggest that, in contrast to highly specific glycan-binding proteins, certain *O*-glycopeptidases may employ a recognition strategy centered on minimal glycan features (e.g. GalNAc) combined with structural permissiveness, enabling function across chemically diverse glycoprotein landscapes.

Together, these findings are consistent with a model in which peptidase_M60 *O*-glycopeptidases contribute to enterococcal ecological versatility by enabling access to *O*-glycosylated proteins across a broad spectrum of biological contexts in which *O*-glycosylated proteins are encountered. The widespread occurrence of peptidase_M60 homologs in enterococci isolated from environmental niches, combined with the broad substrate tolerance and permissive active site architecture demonstrated here, is consistent with the possibility that these enzymes may also support nutrient acquisition in saprophytic settings where complex glycoproteins are available. However, defining the precise substrates and physiological roles of peptidase_M60 enzymes in non-host environments will require future investigation.

## Materials and methods

### Materials

Materials were purchased from Sigma-Aldrich unless otherwise specified.

### Protein production and purification

Gene constructs for the *E. faecium* and *E. faecalis* M60 catalytic modules, EfmM60 and EfcM60, respectively, ordered from GenScript pre-cloned into pET28a. The gene fragments encoded residues 303–816 of MDV4556911 from *E. faecium* strain 51271882 and residues 26–554 of HCR3094972 from *E. faecalis* strain BE16. Protein production and purification by immobilized metal affinity chromatography used procedures identical those used previously (Medley et al. 2022). Purified protein was concentrated in a stirred ultrafiltration unit with a 10 kDa molecular weight cut-off (MWCO) membranes (Millipore). The concentration of each protein in buffer was determined by taking the absorbance at 280 nm and using the predicted extinction coefficient of each protein.

### Mucinase assay

Procedures for the microplate-based mucinase assay were identical to those described previously using biotinylated bovine submaxillary mucin (BSM) (0.08 μg/ml BSM in PBS pH 7.0) or biotinylated T cell immunoglobulin and mucin containing protein 4 (TIM4) (0.08 μg/ml in PBS pH 7.0) (Noach et al. 2017). Bovine serum albumin was used to replace the *O*-glycopeptidases for the negative control and IMPa was used as a positive control. The assay was performed in the absence and presence of 50 μM EDTA. Samples were read on a Spectramax M5 plate reader using SoftMax Pro-6.2.1 software.

### Generation of FRET substrates and confirmation of glycosylation

The FRET substrates were those used previously (Pluvinage et al. 2026). Briefly, substrate constructs comprised in order from the N-terminus, a 6-his tag for purification, the mNeonGreen fluorescent protein domain, an eleven amino acid linker, and C-terminal mScarlet I domain. The linkers represented potentially O-glycosylated mucin and non-mucin like PTS repeats (Supplementary Fig. 4). Unglycosylated and glycosylated FRET substrates were produced as described previously with the glycosylation status confirmed by a combination of lectin blotting and mass spectrometry. The final panel of substrates used included 24 unglycosylated substrates and 44 glycosylated substrates with Tn, C1, 3SC1, C2 or 6SC1 glycans.

### FRET screen

FRET activity screens were performed using methods directly based on those described previously (Canil et al. 2026; Pluvinage et al. 2026). FRET activity screens were setup in 384 well low-profile, clear bottom, black side microplates. 5 μl of each substrate at 0.4 mg/ml (7.2 μM) in reaction buffer (50 mM HEPES, pH 7.0) was dispensed into the tray. Reactions were initiated by the addition of 5 μl of enzyme at double the desired final concentration. Enzyme stocks were in reaction buffer with 200 μM ZnCl_2_. Samples reactions were prepared in duplicate. A paired set of controls were included and contained substrate but only reaction buffer with 200 μM ZnCl_2_ was added. Readings were taken at room temperature (22 °C) by excitation at a wavelength of 430 nm with measurement of emission intensity at 518 nm (F518) and 590 nm (F590) on a SpectraMax M5. Data were processed by first averaging the duplicate reads then calculating the ratio of F518/F590 (F_R_) for samples and matched controls. The ratio of sample and control F_R_ values minus 1 gives ΔFRET (ΔFRET = F_R,sample_/F_R,control_ - 1). This is referred to as the Ratio-of-Ratios or RoR approach. An increase in ΔFRET indicates a decrease in FRET due to cleavage of the substrate. This was displayed as a heat-map vs time with a ΔFRET threshold of 1 (i.e. a doubling of the signal) assigned yellow and values below 0.2 colored black.

### Kinetics

Kinetic experiments were performed using methods directly based on those described previously (Canil et al. 2026; Pluvinage et al. 2026). Individual reactions were setup in a 10 μl final volume using a range of eleven final substrate concentrations from 0.25–6 μM for MUC5AC_R2 or ten concentrations from 0.25–4 μM for MUC16. Reactions were setup using 5 μl of substrates containing 200 μM ZnCl and initiated by the addition of 5 μl of 2× enzyme stocks at concentrations of 0.1 μM and 0.2 μM for EfmM60. A set of matched controls with buffer replacing the enzyme was included. All reaction and control samples were setup in duplicate. Reads were taken with the same parameters as the FRET screen with an ~60 second interval and the total kinetic run time of ~4 hours. The raw fluorescence intensities of the controls were averaged, as were the duplicate reaction measurements. A reaction progress of ΔF vs time for each set of reaction measurements were calculated as (F518-F590)_sample_-(F518-F590)_control_. This resulted in each enzyme concentration giving a set of progress data comprising ten or eleven reactions, for a total of twenty (MUC16) or twenty-two progress (MUC5AC_R2) curves. Using DynaFit, this data was fit with the Van Slyke-Cullen model (Kuzmič 2009) in three different ways, all with global fits: all data together and each enzyme concentration separately.

### Crystallization and structure determination

EfmM60 for crystallization was prepared by removing with 6-his tag with thrombin treatment. Cleaved protein was separated by size exclusion chromatography (SEC) with a S-200 HR column (GE Healthcare) in 20 mM Tris–HCl pH 8.0 and 0.1 M NaCl. Fractions containing purified protein were collected and concentrated using an Amicon stirred filtration unit with a 10 kDa MWCO membrane (Millipore). Crystallization conditions for EfmM60 were screened utilizing the sitting drop method in 96-well crystallization trays and protein at 22 mg/ml with a 1:1 (vol:vol) ratio of screen condition to protein. Crystal trays were stored at 18 °C. Crystal were optimized by the hanging drop method to a final condition of 0.1 M NaCl and 20% w/v PEG 3350. Data was collected on crystal soaked in 8 mM Galβ1–3GalNAcα1-Ser (serinyl-core 1 or C1-Ser, from Biosynth) for 20 minutes and cryoprotected in the crystallization condition supplemented with 20% ethylene glycol.

All X-ray diffaction data were collected at 100 K under a nitrogen cryostream. Diffraction data were collected on an instrument comprising a Pilatus S200K 2D detector, a MicroMax-007HF X-ray generator with a VariMaxTM-HF ArcSec Confocal Optical System and an Oxford Cryostream 800. The HKL2000 suite was utilized to index, scale, and merge the diffraction images (Otwinowski and Minor 1997). The structure of EfmM60 was solved by molecular replacement using PHASER (Mccoy et al. 2007) with a search model generated by AlphaFold 2 (Jumper et al. 2021). The structure was manually corrected in COOT (Emsley et al. 2010) and further refined with Phenix refine (Liebschner et al. 2019). Water molecules in the structures were added utilizing COOT “Find Waters” and manually inspected following Phenix refine. Data collection and final model statistics are summarized in Supplementary Table 2.

## Supplementary Material

EfmM60_paper_supplementary_cwag076

## Data Availability

The atomic coordinates for the two crystal structures reported here have been deposited in the Research Collaboratory for Structural Bioinformatics Protein Databank (www.rcsb.org) under the accession codes PDB ID 35YM.
