# Supplementary material for: Functional conservation of divergent peptidase_M60 O-glycopeptidases in enterococcus
Source: Glycobiology. 2026 Sep 7;36(10):cwag076. doi: 10.1093/glycob/cwag076 (PMC13580078; doi:10.1093/glycob/cwag076)
Supplement: Supplementary file 1 [file efmm60_paper_supplementary_cwag076.docx]

**SUPPLEMENTARY MATERIAL FOR:**

**The genomes of some *Enterococcal* species encode a mucin active *O*-glycopeptidase.**

Liam Mihalynuk^1,3^, Benjamin Pluvinage^1,3^, Olivia Canil^1^, Nicole Thompson^2^, Warren Wakarchuk^2^, and Alisdair B. Boraston^1^*.

^1^Department of Biochemistry and Microbiology, University of Victoria, PO Box 1700 STN CSC, Victoria, British Columbia, V8W 2Y2, Canada.

^2^Department of Biological Sciences, University of Alberta, Edmonton, AB T6G 2E9, Canada.

^3^these authors contributed equally.

Running title: Analysis of enterococcal *O*-glycopeptidases

^*^To whom correspondence should be addressed: Alisdair B. Boraston, Department of Biochemistry and Microbiology, University of Victoria, Victoria, British Columbia, Canada, V8P 5C2; boraston@uvic.ca; Tel. +1 (250) 472-4168;	Fax. +1 (250) 721-8855

#### Supplementary Table 2. X-ray data collection and structural statistics

|  | *Efm*M60 Core 1-Ser |
| --- | --- |
| ***Data Collection*** |  |
| Beamline | Home Beam |
| Wavelength (Å) | 1.54178 |
| Space Group | P1 |
| Cell Dimensions |  |
| *a, b, c* (Å) | 45.7, 82.4, 84.4 |
| α, β, γ (º) | 70.1, 77.3, 80.3 |
| Resolution (Å) | 30.00-2.30 (2.34-2.30) |
| Rmerge | 0.102 (0.492) |
| R_pim_ | 0.051 (0.293) |
| CC1/2 | 0.983 (0.868) |
| <I/σI> | 13.3 (2.1) |
| Completeness (%) | 99.9 (99.4) |
| Redundancy | 3.8 (3.4) |
| No. of reflections | 192518 |
| No. Unique | 50563 |
|  |  |
| ***Refinement*** |  |
| Resolution (Å) | 2.30 |
| Rwork/Rfree | 0.21/0.26 |
| No. of atoms |  |
| Protein | 3917(A), 3959 (B) |
| Ligand | 2 (ZN), 4 (EDO),  32 (C1-ser_A), 32 (C1-ser_B) |
| Water | 197 |
| *B*-factors |  |
| Protein | 34.6 (A), 32.5 (B) |
| Ligand | 40.1 (ZN), 36.7 (EDO),  40.8 (C1-ser _A), 37.2 (C1-ser_B) |
| Water | 31.7 |
| r.m.s.d |  |
| Bond lengths (Å) | 0.004 |
| Bond angles (°) | 0.706 |
| Ramachandran (%) |  |
| Preferred | 97.2 |
| Allowed | 2.7 |
| Disallowed | 0.1 (1 residue) |

Values for highest resolution shells are shown in parenthesis

## Supplementary Figure 1. AlphaFold 3 models of NNE88_11785 and ON393_002825 from *Enterococcus faecium* strain 51271882 and *Enterococcus faecalis* strain BE16, respectively.

## Supplementary Figure 2. Kinetic analysis of EfmM60. A) – C) Progress curves (symbols) and fits (solid lines) on the MUC16 linker substrate for the combined experiments at 0.1 μM and 0.2 μM EfmM60 (A), at only 0.2 μM EfmM60 (B), and at only 0.1 μM EfmM60 (C). D) – E) Progress curves (symbols) and fits (solid lines) on the MUC2_R1 linker substrate for the combined experiments at 0.1 μM and 0.2 μM EfmM60 (A), at only 0.2 μM EfmM60 (B), and at only 0.1 μM EfmM60 (C)

## Supplementary Figure 3. Modeling the extended active sites of EfmM60, EfcM60, and SmE. A) Surface representation of the active site of EfmM60 with C2-Threonine positions by overlap with BcM60C in complex with this ligand (PDB ID 9PLA). B) Surface representation of the AlphaFold 3 model of EfcM60. C) Surface representation of the AlphaFold 3 model of SmE. Putative glycan binding subsites are labeled in all panels.

**Supplementary Figure 4.** Sequences of the linkers used in the panel of FRET substrates. Numbers above the sequences are scores generated by IsoGlyP to indicate the propensity of a certain residue to be an acceptor for *O*-GalNAc by ppGalNAc T2.
